# Epigallocatechin-3-gallate inhibits H_2_O_2_-induced apoptosis in Mouse Vascular Smooth Muscle Cells via 67kD Laminin Receptor

**DOI:** 10.1038/s41598-017-08301-6

**Published:** 2017-08-10

**Authors:** Xue Yan, Yanfei Li, Han Yu, Wei Wang, Chunyan Wu, Yang Yang, Yongjia Hu, Xiujuan Shi, Jue Li

**Affiliations:** 1 Institute of Clinical Epidemiology and Evidence-based Medicine, Tongji University School of Medicine, 1239 Siping Road, Shanghai, 200092 China; 2 Key Laboratory of Arrhythmias of The Ministry of Education of China, Tongji University School of Medicine, 1239 Siping Road, Shanghai, 200092 China; 3 School of Medical Technology, Shanghai University of Medicine & Health Sciences, Shanghai, 201318 China

## Abstract

Epigallocatechin-3-gallate (EGCG) is one of the major polyphenolic compounds present in green tea extracts and has been used as a potential drug for the treatment of numerous diseases. The present study aimed to elucidate the role and mechanism of EGCG in protecting against H_2_O_2_-induced apoptosis in mouse vascular smooth muscle cells (VSMCs). VSMCs were pretreated with various concentrations of EGCG for 2 hours prior to treatment with H_2_O_2_. Treatment with H_2_O_2_ significantly decreased the cell viability and induced apoptosis of VSMCs, which were attenuated by pretreatment with EGCG. In particular, EGCG pretreatment significantly inhibited the H_2_O_2_-induced upregulation of cleaved forms of caspase-3, caspase-8, and caspase-9, Bax, CathepsinD, and downregulation of Bcl-2. Moreover, the antioxidation effect of EGCG on VSMCs was determined to be associated with the 67kD laminin receptor (67LR). Our results demonstrated that EGCG improved cell viability and protected VSMCs against oxidative stress through both extrinsic and intrinsic pathways, while 67LR is likely to be an active and key receptor of EGCG. These findings provide a novel molecular mechanism of EGCG in inhibiting H_2_O_2_-induced apoptosis in VSMCs, as well as its function in preventing the development of atherosclerosis.

## Introduction

Cardiovascular disease (CVD) remains the major cause of morbidity and mortality worldwide^1^. There are several risk factors that contribute to the increased prevalence of CVD including atherosclerosis, which is responsible for nearly half of all deaths in developed countries^2^. Indeed, complications as a result of atherosclerosis can often lead to the increased morbidity and mortality of cardiovascular diseases in general^3^. Therefore, it is important to explore the mechanisms involved in the development of atherosclerosis in order to prevent its occurrence. Atherosclerosis is a typical multifactorial disease, which is dependent on the formation of early atherosclerotic lesions, as well as the oxidative stress and apoptosis of Vascular smooth muscle cells (VSMCs)^4^. VSMCs are located in the medial layer of blood vessels, and are the main type of cell present in the artery^5^. The abnormal apoptosis of VSMCs is associated with numerous pathologies such as inflammation, calcification and thrombosis^6^. Therefore, inhibiting the apoptosis of VSMCs can be an effective preventive strategy in slowing down the generation of plaque and development of atherosclerosis. Hydrogen peroxide (H_2_O_2_), a highly reactive oxygen species (ROS), gives rise to wide spread oxidative damage, and has been widely used to mimic oxidative stress in *vitro* ^7^. Previous studies have demonstrated that H_2_O_2_ may also activate apoptosis *in vivo* ^8^. The mitochondria-mediated intrinsic pathway and the cell death receptors-mediated extrinsic pathway are the main pathways involved in cell apoptosis^9^. These pathways activate distinct apical caspases (caspase-8 or caspase-9) and consequently activate the common downstream executioner caspase-3^10^. Moreover, expression of Bcl-2 has been identified as an important marker of anti-apoptosis in the intrinsic pathway^11^.

Green tea is one of the most popular beverages worldwide, and has long been known to possess various beneficial biological functions^12^. Among the numerous polyphenols that have been isolated from green tea, such as epicatechin (EC), epicatechin-3-gallate (ECG), epigallocatechin (EGC) and epigallocatechin-3-gallate (EGCG), EGCG has been demonstrated to be the main biologically active substance in green tea^13^. Previous epidemiological studies had demonstrated that consumption of green tea was associated with a reduction in the prevalence, as well as the morbidity and mortality associated with numerous diseases such as diabetes, hypertension, cardiovascular disease and various types of cancers^14–18^. Previous studies have demonstrated that the green tea extract EGCG protected cardiomyocytes against ischemia/reperfusion-induced apoptosis both *in vivo* and *in vitro* ^19^. EGCG had also been demonstrated to reduce blood pressure and improve endothelial function in hypertensive rats^20^. In addition, EGCG significantly inhibited atherosclerosis in rabbits^21^, while EGCG was also shown to inhibit ox-LDL induced vascular endothelial dysfunction^22^. Furthermore, EGCG was demonstrated to inhibit various different intracellular pathways, leading to pro-apoptotic and cytotoxic effects in tumor cells^20^. However, little is known about the molecular mechanisms of EGCG’s anti-oxidative activity in H_2_O_2_-induced apoptosis in VSMCs.

The membrane bound 67-kDa laminin receptor (67LR) is a non-integrin cell surface receptor and is frequently over-expressed in tumor cells^23^. 67LR had been previously identified as a cell surface receptor of EGCG in cancer cells, and played a key role in cancer protection^13^. Moreover, 67LR was shown to act as a cancer-specific cell death receptor, and mediated the apoptotic signaling pathway in multiple myeloma cell^24^. Despite the importance of 67LR in regulating EGCG activity in cancer cells, the underlying mechanisms of 67LR as an active receptor of EGCG in mouse VSMCs remains unclear.

In the present study, we examined the role of EGCG in preventing H_2_O_2_-induced apoptosis of VSMCs and verified the role of 67LR as an active and key receptor of EGCG required for preventing H_2_O_2_-induced apoptosis in VSMCs.

## Results

### Effect of EGCG and H_2_O_2_ on cell viability of VSMCs

**Figure 1:**
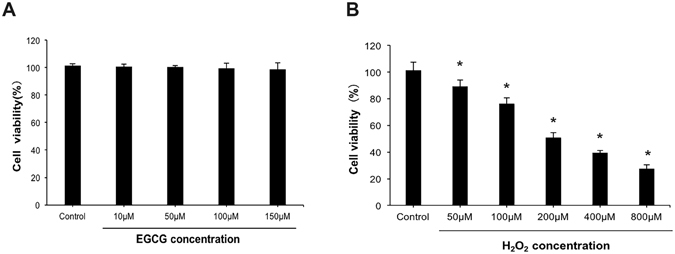
The effect of EGCG on cell viability. (**A**) VSMCs were incubated with 10, 50, 100, or 150 μM EGCG for 2 h and cell viability was assessed using CCK-8 assay. (**B**) CCK-8 assay cytotoxic effect of H_2_O_2_ on VSMCs after incubation with 50, 100, 200, 400, or 800 μM H_2_O_2_ for 30 min. Results were expressed as mean ± S.D.(n = 5). *P < 0.05 vs. control group.

We first examined the antioxidation effect of EGCG following H_2_O_2_-induced oxidative stress using mouse primary VSMCs. The cytotoxic effect of EGCG on VSMCs was determined by treatment with different concentrations (10 μM, 50 μM, 100 μM and 150 μM) of EGCG. There were no significant differences in cell viability between the treatment groups and the control group, which indicated that EGCG had no cytotoxic effect on VSMCs (Fig. 1A). We further determined the concentration-dependent effect of H_2_O_2_ in inducing oxidative stress in VSMCs by treatment with 0 μM, 50 μM, 100 μM, 200 μM, 400 μM or 800 μM H_2_O_2_ for 30 min, respectively. There was a significant dose-dependent decrease in cell viability following incubation with different concentrations of H_2_O_2_ for 30 min. When cells were incubated with 200 μM H_2_O_2_, cell viabilities were attenuated by 50% compared with the control group (Fig. 1B). Hence, 200 μM H_2_O_2_ was selected as the appropriate concentration for inducing oxidative stress in VSMCs in the subsequent experiments.

### The protective effect of EGCG on H_2_O_2_-induced apoptosis

**Figure 2:**
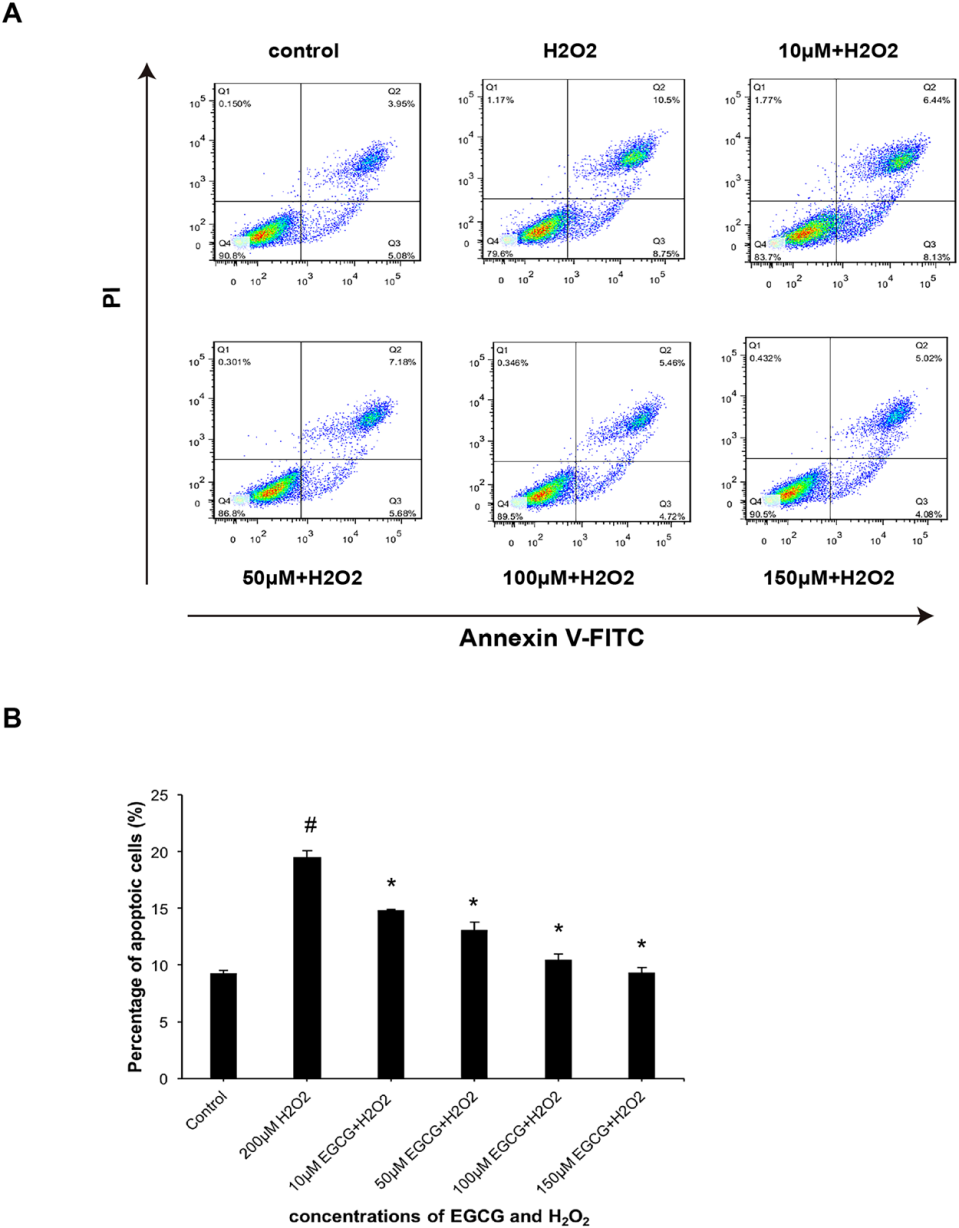
EGCG reduced H_2_O_2_-induced apoptosis in VSMCs. (**A**) VSMCs were pretreated with 10, 50, 100, or150μM EGCG for 2 h, followed by treatment with 200 μM H_2_O_2_ for 30 min. Annexin V-FITC/PI staining and flow cytometry was used to assess the rate of apoptotic cells, which was defined as the sum of early and late apoptosis events. Flowjo software was used to analyze flow cytometry results. (**B**) Statistical data were represented based on experiments performed in triplicate. Results were expressed as Mean ± S.D (n = 5). *P < 0.05 vs. H_2_O_2_ treatment group, ^#^p < 0.05 vs. control group.

We further performed Annexin V-FITC/PI assay to determine whether H_2_O_2_ could also induce VSMCs apoptosis. Following treatment with 200 μM H_2_O_2_ for 30 min, the proportion of apoptotic cells was approximately double that of untreated VSMCs, which demonstrated that H_2_O_2_ successfully induced VSMCs apoptosis (Fig. 2A,B). We therefore investegated whether EGCG pretreatment could protect against H_2_O_2_-induced apoptosis. Annexin V-FITC/PI assay was also utilized to determine the rate of apoptosis based on the sum of early and late apoptosis events. Pretreatment with different concentrations of EGCG significantly decreased the rate of apoptosis induced by 200 μM H_2_O_2_. In particular, pretreatment with 100 μM and 150 μM EGCG almost completely attenuated the rate of H_2_O_2_-induced apoptosis in VSMCs (Fig. 2A,B). Taken together, these results suggested that EGCG had a strong protective effect on H_2_O_2_-induced apoptosis, in a concentration-dependent manner.

### Role of cleaved caspase-3, cleaved caspase-8, cleaved caspase-9, Bcl-2, Bax and Cathepsin D in H_2_O_2_-induced apoptosis

**Figure 3:**
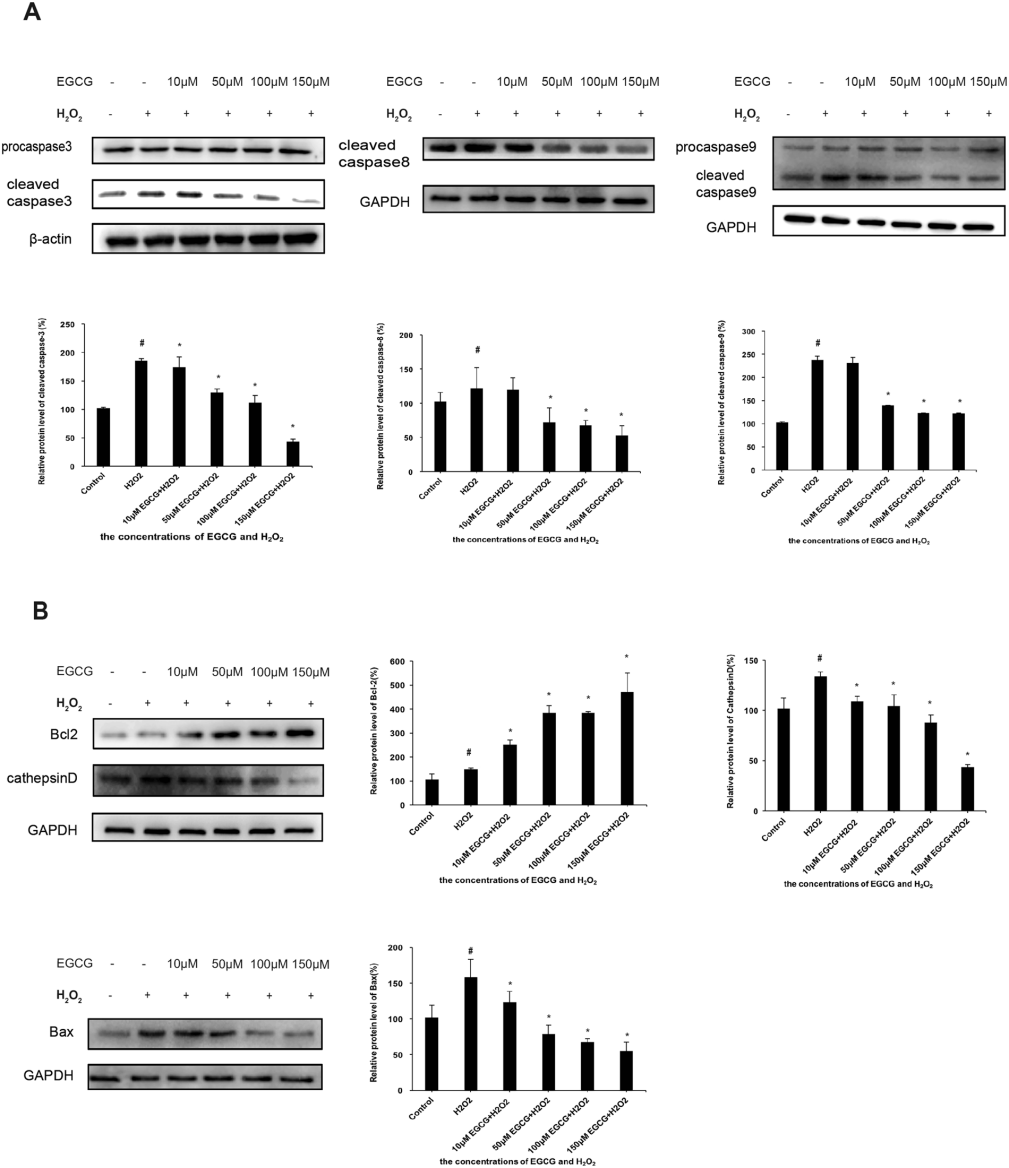
Effect of EGCG on H_2_O_2_-induced changes in protein expression levels of cleaved caspase-3, cleaved caspase-8, cleaved caspase-9, Bcl-2, Bax, and Cathepsin D. (**A**) VSMCs were pretreated with 10, 50, 100, or 150 μM EGCG for 2 h, followed by treatment with 200 μM H_2_O_2_ for 30 min. Data indicated represent ative western blots of caspase-3, caspase-8, caspase-9. (**B**) Representative western blots of Bcl-2, Bax, Cathepsin D and their respective protein expression levels. β-actin and GAPDH were used as internal controls. Quantitative results were expressed as Mean ± S.D. from 5 independent experiments. *P < 0.05 vs. H_2_O_2_ treatment group, ^#^p < 0.05 vs. control group.

We next examined the expression levels of key proteins involved in apoptosis signaling pathway to determine the underlying molecular mechanisms of EGCG in protecting VSMCs from H_2_O_2_-induced apoptosis. The activation of the caspase family were detected by using antibodies specific for the cleaved form of caspase-3, caspase-8 and caspase-9. H_2_O_2_ treatment significantly increased the expression levels of cleaved form of caspase-3, caspase-8 and caspase-9, which indicated that H_2_O_2_ activated the extrinsic apoptosis pathway signaling pathway. Moreover, the expression levels of cleaved caspase-3, cleaved caspase-8 and cleaved caspase-9 were attenuated by pretreatment with EGCG (50, 100, and 150 μM), to almost the level of untreated control group (Fig. 3A). Furthermore, H_2_O_2_ treatment significantly increased the expression levels of Bax and CathepsinD, and decreased the expression of Bcl-2 compared with the control group, which were reversed by pretreatment with EGCG (Fig. 3B). These results suggested that the protective effect of EGCG against H_2_O_2_-induced apoptosis is likely mediated by the caspase family and mitochondrial apoptotic signaling pathways.

### Effect of EGCG on H_2_O_2_-induced changes of 67LR

**Figure 4:**
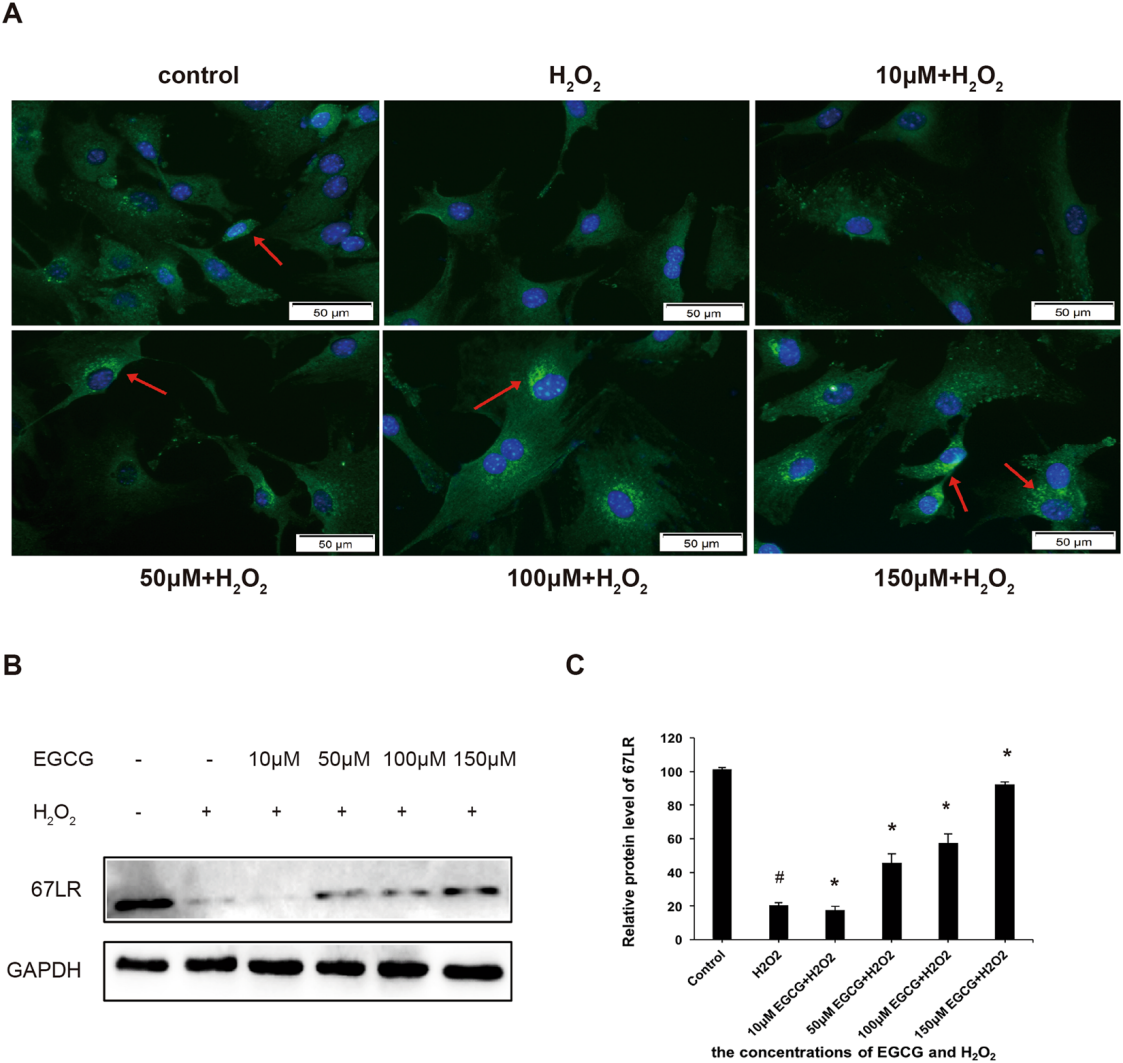
Effect of EGCG on H_2_O_2_-induced changes in 67LR. (**A**) Immunocytochemistry analysis was performed using anti-67LR antibody and Alexa Flouor-488 conjugated secondary antibody(green). The nuclei were stained with DAPI(blue). VSMCs were pretreated with 10, 50, 100, or 150 μM EGCG for 2 h, followed by treatment with 200 μM H_2_O_2_ for 30 min. (**B**) Western blot showing the expression of 67LR after treatment with EGCG and H_2_O_2_. Results were expressed as Mean ± S.D. from 5 independent experiments. *P < 0.05 vs. H_2_O_2_ treatment group, ^#^p < 0.05 vs. control group.

We also investigated whether there were changes in the expression of 67LR, as an active receptor of EGCG, following H_2_O_2_-induced apoptosis in VSMCs. Immunofluorescence microscopy revealed that 67LR was abundantly expressed on the surface of VSMCs in the untreated control group. However, H_2_O_2_ treatment significantly inhibited 67LR expression, which was effectively prevented by pretreatment with EGCG in a concentration-dependent manner (Fig. 4A). Furthermore, western blot analysis revealed that the protein expression level of 67LR following H_2_O_2_-induced apoptosis was consistent with our previous results (Fig. 4B). Thus, these results suggested that 67LR is down regulated to a lesser extend with increasing EGCG concentration in VSMCs.

### The protective effects of EGCG on H_2_O_2_-induced apoptosis following shRNA silencing of 67LR

**Figure 5:**
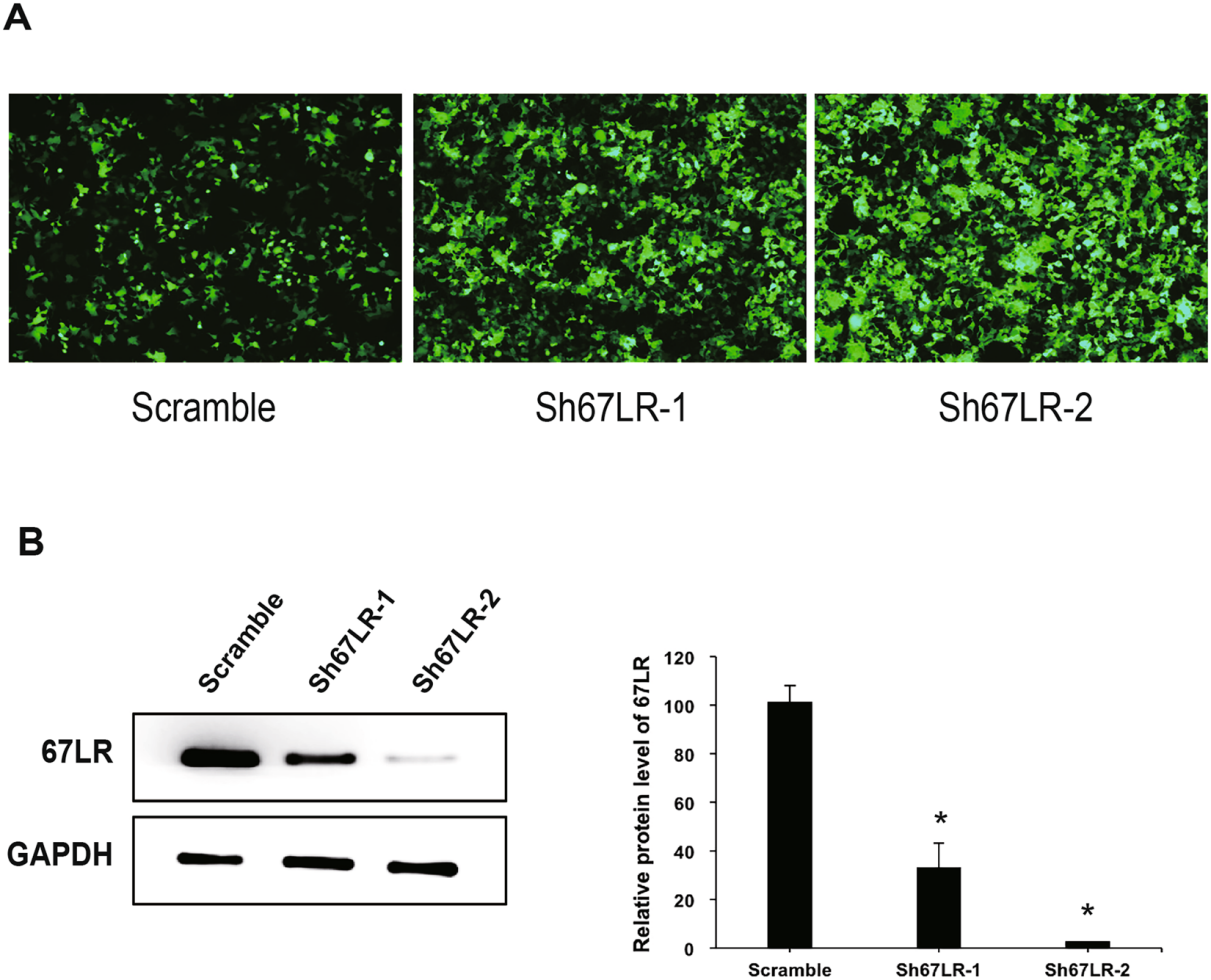
The efficiency of shRNA silencing of 67LR. (**A**) Transfection efficiency in 293 T cells. (**B**) Western blot analysis showing the expression of 67LR after silencing by shRNA plasmid. *p < 0.05 vs. Scramble group.

To further verify that 67LR was an active receptor of EGCG required for its protection against H_2_O_2_-induced apoptosis, we utilized lentivirus mediated RNAi technology to silence 67LR in VSMCs. Firstly, the 67LR short hairpin RNA (shRNA) targets (sh67LR-1 and sh67LR-2) were cloned into the lentivirus vectors, and subsequently the lentivirus packing plasmids and vector plasmids which target 67LR were co-transfected into 293 T cells. The transfection efficiency of sh67LR-1 and sh67LR-2 were greater than 80% and 90%, respectively (Fig. 5A). Next, VSMCs were infected with lentivirus which expressed either the 67LR targeted shRNA or a scrambled control shRNA. Western blot analysis confirmed that the expression levels of 67LR were significantly decreased by 75% and 95% following transfection with sh67LR-1 and sh67LR-2, respectively, compared to the scrambled control shRNA (Fig. 5B).

**Figure 6:**
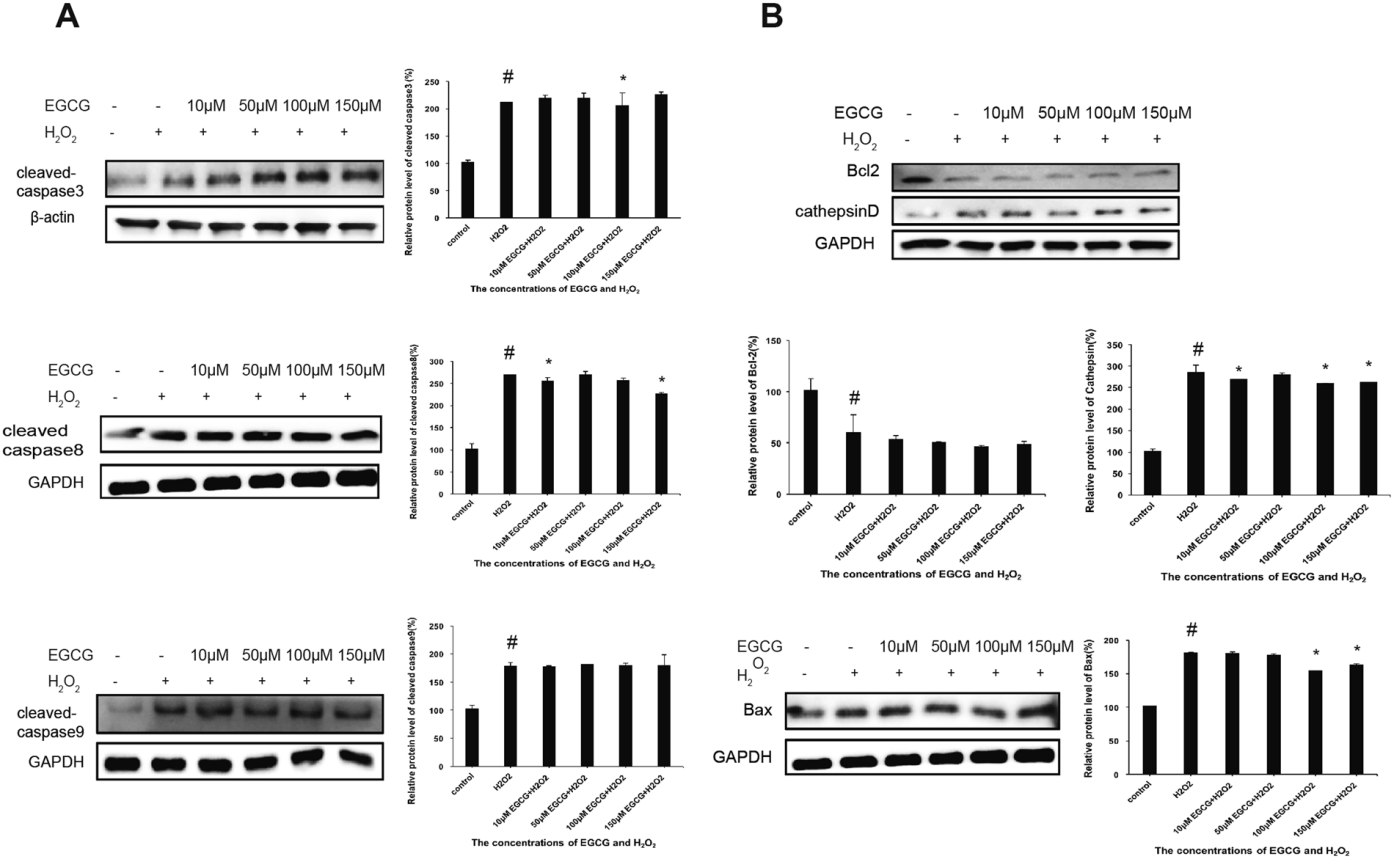
The protective effects of EGCG on H_2_O_2_-induced apoptosis following shRNA silencing of 67LR. (**A**) Western blot analysis showing the expression levels of caspase-3, caspase-8 and caspase-9 following shRNA silencing of 67LR, and subsequent pretreatment with 10, 50, 100, or 150 μM EGCG for 2 h, followed by treatment with 200 μM H_2_O_2_ for 30 min. (**B**) Western blot analysis showing the expression levels of Bcl-2, Bax and Cathepsin D following shRNA silencing of 67LR, and subsequent pretreatment with 10, 50, 100, or 150 μM EGCG for 2 h, followed by treatment with 200 μM H_2_O_2_ for 30 min. β-actin and GAPDH were used as internal controls. Results were expressed as Mean ± S.D. from 5 independent experiments. *P < 0.05 vs. H_2_O_2_ treatment group, ^#^p < 0.05 vs. control group.

Additionally, following shRNA silencing of 67LR, VSMCs treated with H_2_O_2_ had significantly increased levels of cleaved caspase-3, cleaved caspase-8 and cleaved caspase-9 expression, but was not inhibited by pretreatment with EGCG (Fig. 6A). Moreover, following shRNA silencing of 67LR, pretreatment with EGCG also did not reverse the H_2_O_2_-induced changes in Bcl-2, Bax and CathepsinD expressions (Fig. 6B). Taken together, these results revealed that 67LR is likely to be an active receptor of EGCG required for its protection against H_2_O_2_-induced apoptosis in VSMCs.

## Discussion

EGCG’s ability to protect against atherosclerosis is often associtated with its antioxidative, anti-inflammatory, antiproliferative, antimigration, and antithrombotic properties on VSMCs^25^. Atherosclerosis is a multifactorial cardiovascular disease, which can arise due to oxidative stress and formation of early atherosclerotic lesions. Oxidative stress plays an important role in the pathogenesis of atherosclerosis by promoting plaque formation^26^. Oxidative stress has been shown to stimulate the proliferation, migration and apoptosis of VSMCs during atherosclerosis^27^. Therefore, elucidating the potent antioxidant effect of EGCG on H_2_O_2_-induced apoptosis in VSMCs is important for the prevention of oxidative stress-induced apoptosis.

We first investigated the effect of EGCG on cell viability in VSMCs. There were no significant differences in cell viability between the treatment groups and the control group, which indicated that EGCG had no cytotoxic effect on VSMCs. Pretreatment with EGCG for 48 h prior to H_2_O_2_ treatment significantly improved the survival of mouse muscle cells^28^. However, a previous study revealed the dose-dependent decrease of cell viability following treatment with high concentrations of EGCG (1050 μM) in VSMCs^29^. H_2_O_2_ has been shown to decrease cell ability and induce cell apoptosis in VSMCs^30^. Thus, in the present study, we used H_2_O_2_ treatment for 30 min to establish the apoptosis model in VSMCs.

Apoptosis is mediated by both extrinsic and intrinsic pathways and plays key roles in maintaining normal cell physiology^31^. Inadequate, excessive, or inappropriate apoptosis can all contribute to disease occurrence. H_2_O_2_ induces severe intracellular oxidative stress, which leads to widespread intracellular damage and apoptosis^32^. In the present study, we demonstrated that the apoptosis rate of VSMCs increased significantly following H_2_O_2_ treatment, which was significantly attenuated by pretreatment with 10 μM-150 μM EGCG. EGCG was shown to regulate the apoptotic process in different ways depending on the concentration, type of cell or pathological process^33^. In the present study, we showed that the EGCG-mediated decrease in VSMCs apoptosis was due to the decreased expression of cleaved form of caspase-3, caspase-8 and caspase-9. The caspase family are activated by cleavage at specific aspartic residues following external stimuli, thereby resulting in apoptosis^34^. The intrinsic apoptosis pathway is involved in the transduction of apoptotic stimuli, such as hypoxia, oxidative stress, DNA damage, inadequate nutrients and chemical toxins, which ultimately triggers the release of anti-apoptotic proteins^35^. Apoptotic signals are transduced to the mitochondria through two classes of anti-apoptotic Bcl-2 proteins^36^. We showed that pretreatment with EGCG resulted in the decreased expression of Bax and CathepsinD, as well as the increased expression of Bcl-2. EGCG could affect apoptosis by modulating the level of expression of anti-apoptotic Bcl-2 or pro-apoptotic Bax protein^37, 38^. EGCG has been shown to attenuate oxidative stress in human VSMCs via activation of heme oxygenase-1^39^. In addition, EGCG can relieve oxidative stress by inhibiting endothelin-1-stimulated generation of C-reactive protein in VSMCs^40^. In the present study, we demonstrated that EGCG strongly protected against H_2_O_2_-induced VSMCs apoptosis via activation of caspase family and Bcl-2 signaling pathways. These results revealed that EGCG could modulate both the extrinsic and intrinsic apoptotic pathways by decreasing the expression of cleaved caspase-3, caspase-8, caspase-9, Bax, and CathepsinD, while increasing the expression of Bcl-2.

67LR was shown to be expressed on the surface of normal cells, but overexpressed in several cancer cells^41^. 67LR acted as an EGCG receptor and exerts an inhibitive effect on cancer cell growth^42^. This suggests that EGCG may specifically target cancer cells. However, in our present study, we showed that 67LR was abundantly expressed on the surface of VSMCs, which to our knowledge had never been previously reported. The expression of 67LR was significantly decreased following H_2_O_2_ treatment, but was down regulated to a lesser extent with increasing EGCG concentration. Previous evidence has suggested that the potential action of EGCG in the prevention of cardiovascular diseases is mediated by 67LR^43^. Therefore, we proposed that 67LR plays a key role in EGCG-mediated H_2_O_2_-induced apoptosis in VSMCs. Lentivirus mediated RNAi technology was used to silence 67LR in VSMCs. After silencing, VSMCs treated with H_2_O_2_ had significantly increased levels of cleaved caspase-3, cleaved caspase-8 and cleaved caspase-9 expression, but was not inhibited by pretreatment with EGCG compared with H_2_O_2_ group. Furthermore, pretreatment with EGCG also did not reverse the H_2_O_2_-induced changes in Bcl-2, Bax and CathepsinD expressions. These results indicated that 67LR acts as an active receptor of EGC, thereby mediating the apoptosis signaling pathway in VSMCs.

In summary, we demonstrated that the protective effect of EGCG on H_2_O_2_-induced VSMCs apoptosis was due to the inhibition of caspase family and Bcl-2 signaling pathways, and was mediated by 67LR, a key active receptor of EGCG. Our results also suggested that the consumption of catechins found in green tea is beneficial for the prevention of atherosclerosis and cardiovascular diseases.

## Methods

### Antibodies

Primary antibody against caspase-3 was obtained from Santacruz biotechnology (CA, USA) (#SC-7148). Primary antibodies against caspase-8 (#13423-1-AP), caspase-9 (#10380-1-AP), Bcl-2 (#12789-1-AP), Bax (#50599-2-Ig), CathepsinD (#21327-1-AP), 67LR (#14533-1-AP), GAPDH (#10494-1-AP) and β-actin (#20536-1-AP) were purchased from Proteintech (Chicago, IL, USA). Anti-rabbit secondary antibodies (#SA00001-1) and anti-mouse secondary antibodies (#SA00001-1) were also purchased from Proteintech (Chicago, IL, USA).

### Chemicals and reagents

Dulbecco’s modified Eagle’s medium (DMEM) was purchased from Hyclone (#SH30243.01, Logan, USA) and fetal bovine serum (FBS) was purchased from Gibco (#10099141, New York, USA). Penicillin-Streptomycin Solution was purchased from Hyclone ((#SV30010, Logan, USA). EGCG was obtained from Sigma-Aldrich(#E4268, MO, USA). H_2_O_2_ was purchased from Sangon Biotech (#H1976-500ml, Shanghai, China). Cell Counting Assay kit-8 (CCK-8) was purchased from Dojindo Molecular Technologies (#CK04, MD, Japan). Annexin V-FITC/PI apoptosis assay kit was purchased from CW biotech (#CW2574S, Beijing, China).

### Cell culture and treatment

VSMCs were separated from abdominal aorta of mice and the subculture cells at passages 3–8 were used in all the experiments. VSMCs were cultured in DMEM supplemented with 10% FBS and 1% penicillin and streptomycin in a humidified chamber with 5% CO_2_ at 37 °C. VSMCs were pre-treated with 0 μM, 10 μM, 50 μM, 100 μM or 150 μM EGCG for 2 h, washed with PBS two times and then exposed to 200 μM H_2_O_2_ for 30 min in serum-free DMEM. All experiments were performed 5 times. All animal procedures were performed according to approved protocols from the Institutional Animal Care and Use Committee of Tongji University.

### Cell viability assay

Cell viability was measured using Cell Counting Assay Kit-8 (CCK-8), according to the manufacturer’s instructions. Briefly, 100 μl of VSMCs were seeded into 96-well plates at a density of 1 × 10^4^ cells/well for 24 h, and then treated with different concentrations of EGCG for 2 h at 37 °C. Finally, the absorbance at 450 nm in each well was measured using Microplate Reader (Thermo Fisher Scientific, Inc., Waltham, MA, USA), prior to incubation with 10 μl CCK-8 solution for 3 h. Similarly, 0 μM, 50 μM, 100 μM, 200 μM, 400 μM or 800 μM H_2_O_2_ were added per well to determine the effect of H_2_O_2_ on cell viability. Results were represented as the percentage of the control group.

### Apoptosis detection

VSMCs apoptosis was detected using Annexin V-FITC/PI Apoptosis Detection Kit, according to the manufacture’ protocols. Briefly, VSMCs were cultured in 6-well plates at a density of 1 × 10^5^ cells/well at 37 °C and harvested by EDTA free trypsin for 2 min. Cells were then centrifuged at 1000 × g for 3 min, and washed twice with ice cold PBS prior to resuspension in 250 μl Binding Buffer. VSMCs were incubated with 5 μl Annexin V/FITC and 10 μl PI for 15 min in the dark at room temperature. BD Biosciences FACSCalibur flow cytometer (Franklin Lakes, NJ, USA) was used to analyze the stained samples. Results were represented as the sum percentage of early apoptosis and advanced apoptosis.

### Western blot analysis

Total cell lysates were extracted using ice cold lysis buffer containing 1% phenylmethylsulfonyl fluoride (PMSF). Cells were centrifuged at 12000 × g for 15 min at 4 °C and the resulting supernatants were collected. Total protein concentrations were determined using Bicinchoninic Acid (BCA) kit (Beyotime, Shanghai, China). Protein samples were separated on 10% SDS-PAGE gels and then transferred onto Polyvinylidene-Fluoride (PVDF) membranes (Milipore, MA, USA) with transfer buffer. PVDF membranes were then blocked using 5% non-fat dry milk in Tris-buffered saline containing 1% Tween-20 (TBST) for 1 h at room temperature, then washed 3 times with TBST prior to incubation with primary antibody at 4 °C overnight. Following washing, the membranes were incubated with secondary antibody at room temperature for 1 h. After washing for 3 times with TBST, enhanced chemiluminescence was used to detect the proteins immunoreactive bands. The images were analyzed using Image J. All original full length Western Blot pictures were presented in the Supplemental Figure 1 to Supplemental Figure 4.

### Immunocytochemistry (ICC)

VSMCs cells were fixed with 4% paraformaldehyde for 20 min and rinsed three times with PBS. Subsequently, cells were incubated with blocking solution (PBS containing 1% bovine serum albumin, 0.4% Triton X-100 and 4% normal goat serum) for 60 min, and then incubated with primary anti-67LR antibody (1:1000) at 4 °C overnight. After washing twice, VSMCs were incubated with Alexa Flouor-488 conjugated secondary antibody (Invitrogen, Carlsbad, CA) (1:600, goat anti-rabbit) for 1 h at room temperature. Finally, cells were washed 3 times with PBS, then counterstained with 4′,6-diamidino-2-phenylindole (DAPI) (Vector Laboratories Inc, Burlingame, CA). The expression of 67LR was examined using a fluorescence microscope (OLYMPUS, Japan).

### Lentiviral infection

The 67LR-targeted ShRNA-67LR-1 and ShRNA-67LR-2 sequences were obtained from Sigma-Aldrich (Table 1). The shRNA-67LR sequences were ligated into the PLVX-shRNA2 vector, which expressed a classic scrambled shRNA and green fluorescent protein (ZsGreen). For viral packaging, 293 T cells were cotransfected with lentiviral plasmids using Lipofectamine 2000 (Invitrogen, CA, USA) to generate the recombinant lentivirus. Cell transfection efficiency was observed using fluorescence microscopy after collection of virus supernatant. VSMCs were infected with virus supernatant and the extent of 67LR knockdown was determined using western blot analysis. shRNA-scrambled plasmid was used as a control.

**Table 1:** The 67LR shRNA knockdown sequences.

Name	Sequences
shscramble-F	gatccCAGCGCTGACAACAGTTTCATCTCGAGATGAAACTGTTGTCAGCGCTGTTTTTg
shscramble-R	aattcAAAAACAGCGCTGACAACAGTTTCATATATCTCATGAAACTGTTGTCAGCGCTGg
sh67LR 1 -F	gatccGGAGTGACGGTATCTACATCATACTCGAGTATGATGTAGATACCGTCACTTTTTTTg
sh67LR 1 -R	aattcCAAAAAAAGTGACGGTATCTACATCATACTCGAG TATGATGTAGATACCGTCACTCCg
sh67LR 2 -F	gatccCGGGGAGGTCATGCCTGATCTTTACTCGAGTAA AGATCAGGCATGACCTCCTTTTTTg
sh67LR 2 -R	aattcAAAAAAGGAGGTCATGCCTGATCTTTACTCGAG TAAAGATCAGGCATGACCTCCCCg

### Statistical analysis

All experimental data were expressed as mean ± S.D. Variables between groups were compared using one-way ANOVA, and Tukey’s test for multiple comparisons. Differences were considered to be statistically significant when p < 0.05.

## Electronic supplementary material

Supplementary Information
