# Supplementary material for: Epigallocatechin-3-gallate inhibits H2O2-induced apoptosis in Mouse Vascular Smooth Muscle Cells via 67kD Laminin Receptor
Source: Sci Rep. 2017 Aug 10;7:7774. doi: 10.1038/s41598-017-08301-6 (PMC5552808; doi:10.1038/s41598-017-08301-6)
Supplement: Supplementary file 1 — Supplementary Information [file 41598_2017_8301_MOESM1_ESM.pdf]

Supplement Information

Title：Epigallocatechin-3-gallate inhibits H_2O_2-induced apoptosis in Mouse Vascular Smooth Muscle Cells via 67kD Laminin Receptor

Xue Yan[1,2], Yanfei Li[3], Han Yu[1,2], Wei Wang[1,2], Chunyan Wu[1,2], Yang Yang[1,2], Yongjia Hu[1,2], Xiujuan Shi[1,2,*], and Jue Li[1,2,*]

[1]Institute of Clinical Epidemiology and Evidence-based Medicine, Tongji University School of Medicine, 1239 Siping Road, Shanghai, 200092, China

[2]Key Laboratory of Arrhythmias of The Ministry of Education of China, Tongji University School of Medicine, 1239 Siping Road, Shanghai, 200092, China

[3]School of Medical Technology, Shanghai University of Medicine & Health Sciences, Shanghai, 201318, China

[*]Correspondence to:

Xiujuan Shi, E-mail: xiujuansh@tongji.edu.cn

Jue Li, E-mail: jueli@tongji.edu.cn

Figure1

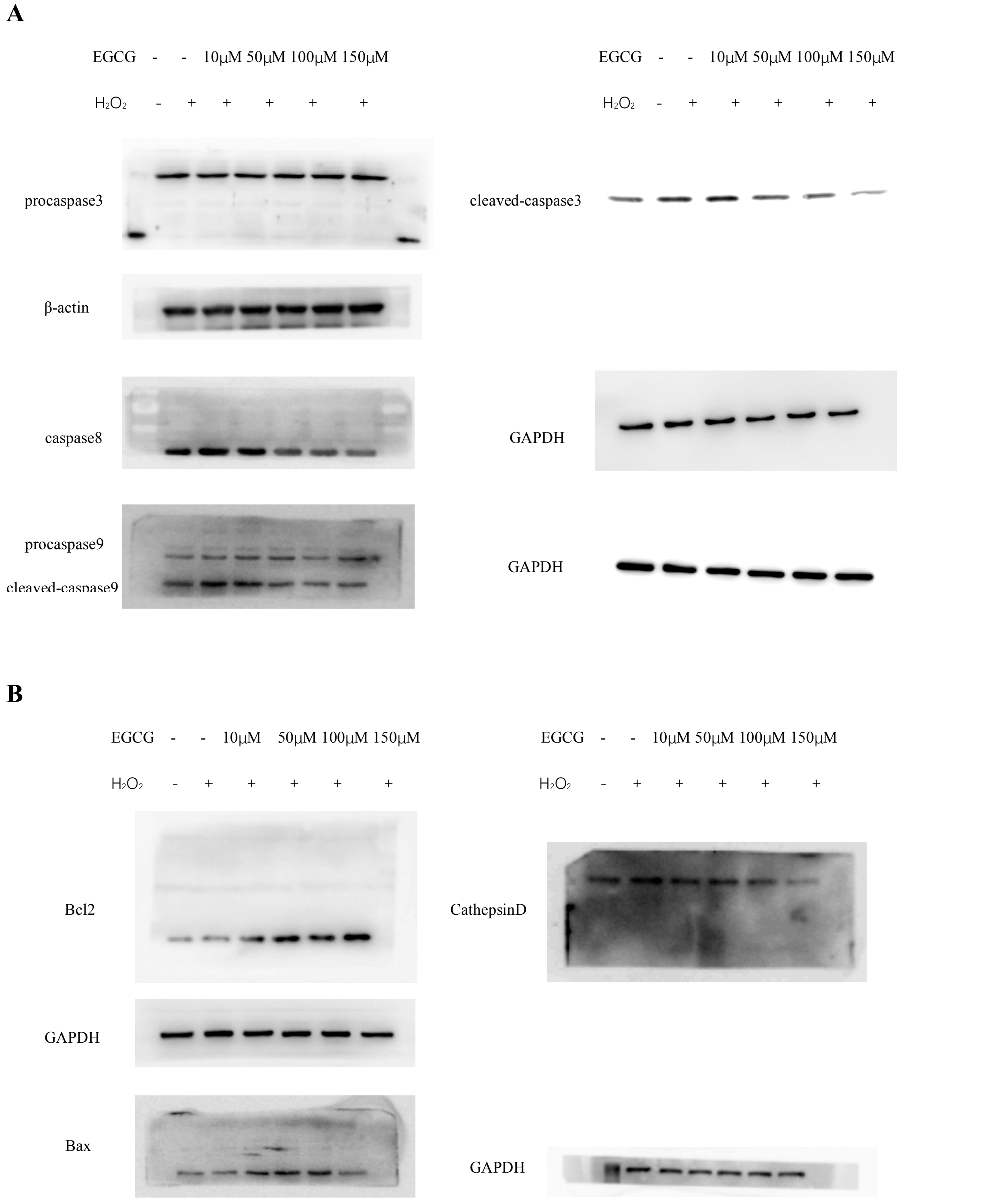

Supplemental Figure1. Full length Western blot used for Figure 3.

Figure2

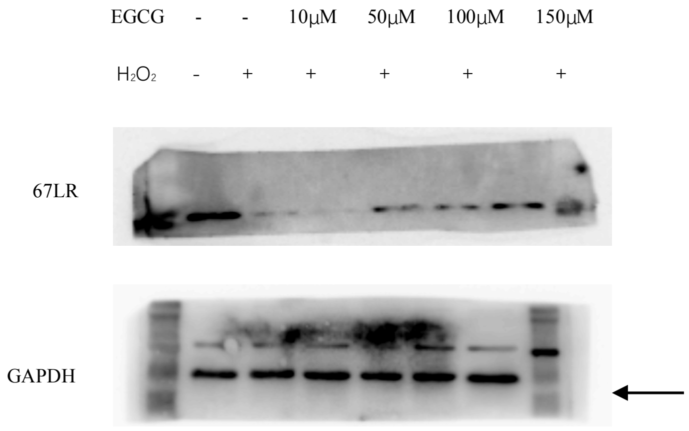

Supplemental Figure2. Full length Western blot used for Figure4.

Figure3

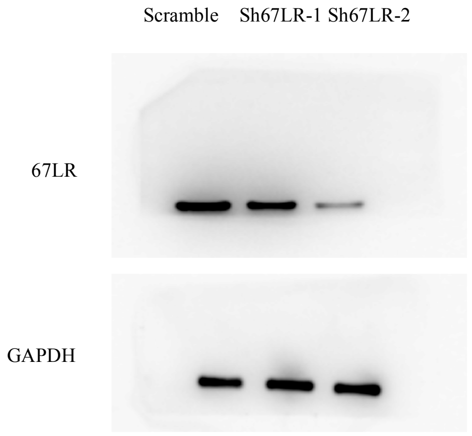

Supplemental Figure3. Full length Western blot used for Figure5.

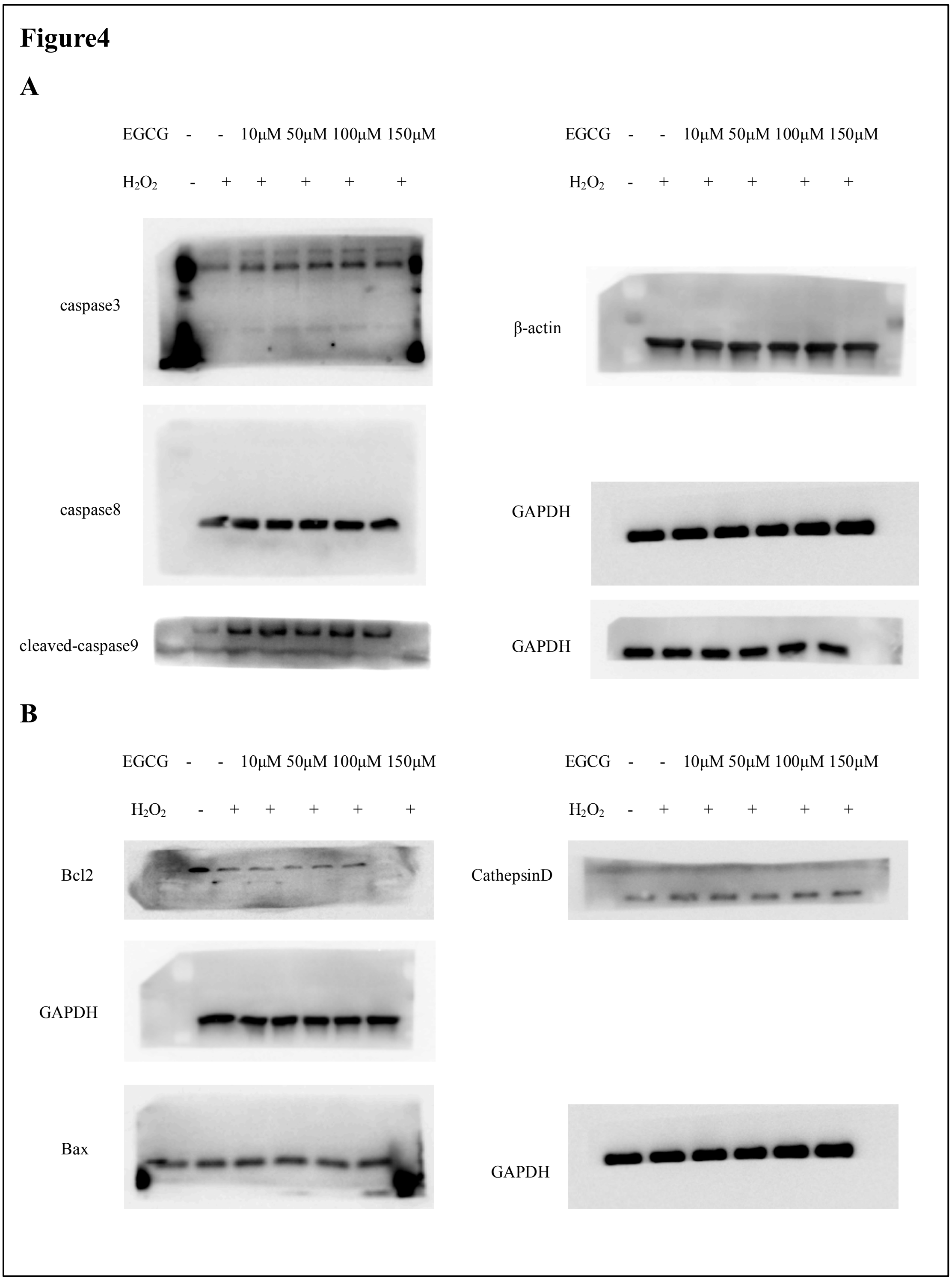

Supplemental Figure4. Full length Western blot used for Figure6.
